# Stress and stability comparison between different systems for high tibial osteotomies

**DOI:** 10.1186/1471-2474-14-110

**Published:** 2013-03-25

**Authors:** Chu-An Luo, Shi-Yuan Hua, Shang-Chih Lin, Chun-Ming Chen, Ching-Shiow Tseng

**Affiliations:** 1 Department of Mechanical Engineering, National Central University, Taoyuan, Taiwan; 2 Graduate Institute of Biomedical Engineering, National Taiwan University of Science and Technology, No. 43, Sec. 4, Keelung Road, Taipei, 106, Taiwan; 3 Department of Orthopedic Surgery, Tri-Service General Hospital, Taipei, Taiwan

**Keywords:** Knee, High tibial osteotomy, Finite-element analysis, Locking screw, Tibial plate

## Abstract

**Background:**

High tibial osteotomy (HTO) with a medial opening wedge has been used to treat medial compartment osteoarthritis. However, this makes the proximal tibia a highly unstable structure and causes plate and screws to be the potentials sources for mechanical failure. Consequently, proper design and use of the fixation device are essential to the HTO especially for overweight or full weight-bearing patients.

**Methods:**

Based on the CT-based images, a tibial finite-element model with medial opening was simulated and instrumented with one-leg and two-leg plate systems. The construct was subjected to physiological and surgical loads. Construct stresses and wedge micromotions were chosen as the comparison indices.

**Results:**

The use of locking screws can stabilize the construct and decrease the implant and bone stresses. Comparatively, the two-leg design provides a wider load-sharing base to form a force-couple mechanism that effectively reduces construct stresses and wedge micromotions. However, the incision size, muscular stripping, and structural rigidity are the major concerns of using the two-leg systems. The one-leg plates behave as the fulcrum of the leverage system and make the wedge tip the zone of tension and thus have been reported to negatively affect the callus formation.

**Conclusions:**

The choice of the HTO plates involved the trade-off between surgical convenience, construct stability, and stress-shielding effect. If the stability of the medial opening is the major concern, the two-leg system is suggested for the patients with heavy load demands and greater proximal tibial size. The one-leg system with locking screws can be used for the majority of the patients without heavy bodyweight and poor bone quality.

## Background

High tibial osteotomy is a surgical treatment for the correction of medial compartment osteoarthritis. In the literature, there are two HTO types, namely laterally closing and medially opening osteotomies [1-4]. The medially opening HTO has become more popular with its better outcome and numerous complications can be avoided [2,5,6]. From the biomechanical viewpoint, the wedge makes the proximal tibia highly unstable, and structural stiffness of the HTO fixation has been reported to be closely related to the postoperative outcome [2,7,8].

Historically, some internal and external fixation devices were used to maintain the instrumented graft in place and stabilize the osteotomized construct [2,5,8,9]. Among the various devices, the Puddu and TomoFix plate systems were the most common type specifically designed for the HTO procedure [1,6,7,10]. However, recent reports have shown that some shorter systems (*e.g.* Puddu system) result in graft nonunion and implant failure [5,11-13]. The clinical study by Nelissen *et al.*[11] revealed that a shorter plate and nonlocking screws provide less ability to stabilize the osteotomized construct. For the TomoFix system with a longer plate, Kolb *et al.*[14] revealed that the use of the locking screws would yield better short-term results. Although a high rate of satisfactory outcome was reported, the improper selection and use of the implant often induced nonunion and even fracture of the HTO construct, especially for overweight or full weight-bearing patients [14-18].

**Figure 1:**
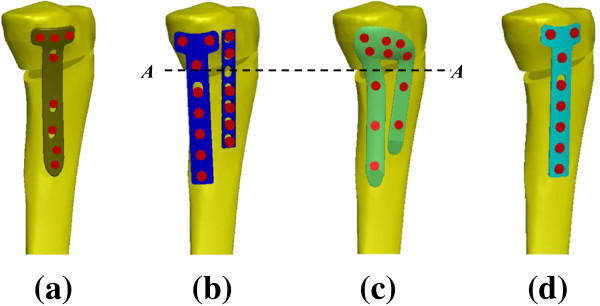
**Four HTO plates used in this study.** (**a**) TomoFix plate. (**b**) T+I plate. (**c**) π plate. (**d**) T plate. The section plane *AA* was denoted to calculate the loads through the anterior and posterior legs of the T+I and π plates.

The TomoFix system is the one-leg design such that the knee loads are transferred into the tibial diaphysis through a single straight plate (Figure 1a). Of all human joints, the knee is one of the most loaded joints and the osteotomized tibia makes the knee structurally unstable. The current authors purposed the concept that the increase of the load-sharing base (*e.g.* two-leg design) below the opening wedge can effectively stabilize the HTO construct. This can be done by the hybrid use of the conventional T and DCP (I-shaped) plate (Figure 1b). The T plate was used at the medioposterior side to stabilize the most heavily loaded region of the knee joint. The I plate was fixed at the anterior region to further support the wedged tibia. However, the screws of both the T and I plates were nonlocking screws. The authors hypothesize that the use of the locking screws and the integration (*i.e*. π-shape) of both T and I plates can increase the structural stability of the bone-plate-screw construct (Figure 1c). The biomechanical comparison between the TomoFix, T+I, and π systems is of bioengineering significance and was the motivation of this study. Using the finite element method, this study compared the stress and stability differences between the one- and two-leg HTO plate systems. The effects of plate legs and locking screws were examined in terms of construct stress and wedge micromotion. The outcome of this study provides design information about the ideal plate for HTO surgery.

## Methods

### Tibia-plate-screw models

**Figure 2:**
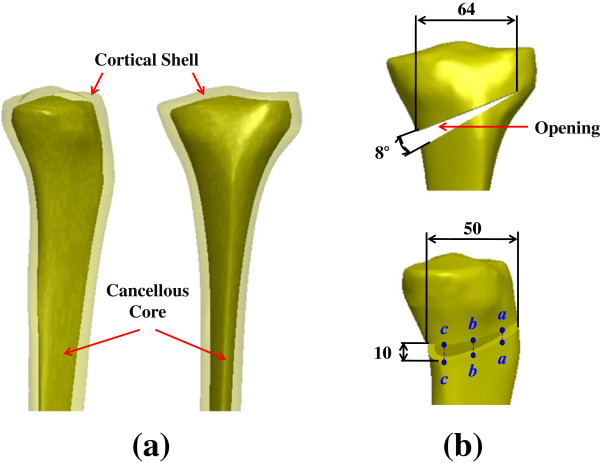
**CT-based tibial model for finite-element analysis: (a) The model consists of cortical shell and cancellous core.** (**b**) Specifications of medial opening (unit: mm and degree). Three edges aa, bb, and cc across the opening were defined to calculate changes in height before and after weight bearing.

A middle-aged male volunteer without any knee disease participated in the scans of the computed tomography (CT) for his left knee. The medical ethics committee, National Taiwan University of Technology and Science, approved the study design. Written informed consent was obtained from all the patients for participation in the study and publication of accompanying images. The CT-scanning images of the tibia with 1-mm slice separation were reconstructed three-dimensionally as a tibial model with triangular surface meshes using the software PhysiGuide, Version 2.3.1 (Pou Yuen Technology Co., LTD, Changhua, Taiwan). The surface model of the proximal tibia was further transformed into a solid model with smooth and seamless surfaces by the software SolidWorks, Version 2010 (SolidWorks Corporation, Concord, MA, USA). The tibial model consisted of a cortical shell and a cancellous core (Figure 2a). The creation of an opening wedge on the medial side was guided by a clinician to simulate a high tibial osteotomy. The characteristic specifications of the wedge size and correction angle were cited from the literature studies [19,20] and shown in Figure 2b.

There were two types of the HTO plate systems used in this study (Figure 1). The first type was the one-leg design: the TomoFix plate and the conventional T plate (Figure 1a and 1d). The second type was the two-leg design: the hybrid use of two conventional T and I plates and the π-shaped plate (Figure 1b and 1c). The π-shaped plate slightly extended the medioposterior area to stabilize the opening in that region. Below and above the opening site, the numbers of the screws to fix the four plates are shown in Figure 1. All plates and screws were developed using the software SolidWorks Version 2010 (SolidWorks Corporation, Concord, MA, USA). The aim of this study was investigate the effect of plate design on construct behavior, thus modeling of the screw threads was omitted to simulate a rigid-bond at the tibia-screw interface.

### Finite-element analysis

The constitutive laws for the bones (cortical and cancellous) were assumed to be linearly elastic, homogeneous, and isotropic. The simulation of bone qualities were base on a middle-aged population, and the values of Young’s modulus and Poisson’s ratio were 17 GPa and 0.33 for cortical bone and 5 GPa and 0.33 for trabecular bone, respectively [21,22]. Except for the titanium-based TomoFix system, the material of all plate systems were surgical stainless steel (AISI 316 L) with homogeneous and linear properties (elastic modulus = 210 GPa, yielding strength = 750 MPa, and Poisson’s ratio = 0.3). The length, width, and thickness of the plates are about 115 × 35 × 3 mm for the TomoFix plate, 115 × 34 × 3 mm for the T plate, 90 × 11 × 3 mm for the I system plate, and 120 × 45 × 3 mm for the π-shape plate, respectively. In this study, the bone graft was neglected in the finite-element analysis to simulate the worst-case scenario of implant loading. Both tibia-plate and plate-screw interfaces were modeled as surface-surface contact elements which allow for separation and slippage. The tangential friction law was based on Coulomb’s criterion, ignoring any friction from adherence with the friction coefficient assumed to be 0.3.

**Figure 3:**
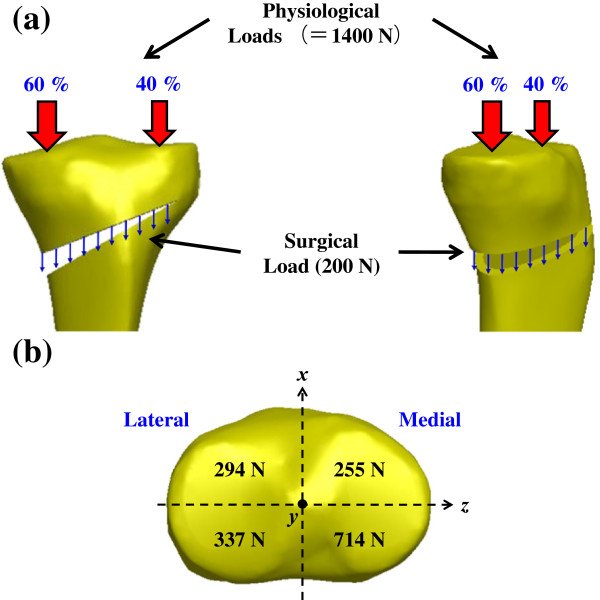
**Schematic diagrams of loads assumed in finite-element models: (a) Physiological (=1400 N) and surgical (200 N) loads were applied to the bone-plate construct.** (**b**) The components of the physiological and surgical loads on the four regions of the tibial plateau.

The interface between the cortical and trabecular bone was assumed to be bonded. The locking screws of both π and TomoFix systems were simulated to rigidly bond with the plate holes. Comparatively, interfacial movement was allowed for the nonlocking screws within the plate holes of the two other configurations. At the proximal portion, the locking screws are 65 mm in length and 5.0 mm in diameter and the nonlocking screws are 65 mm in length and 4.5 mm in diameter. At the distal portion, the locking screws are 36 mm in length and 5.0 mm in diameter and the nonlocking screws are 36 mm in length and 4.5 mm in diameter. Two types of loads were applied to the osteotomized tibial models, including the intervention-induced compression during surgery and physiological loads (bony and muscular) after surgery (Figure 3a). The intervention-induced loads constituted the stabilizing force on the graft that is mainly attributed to the distraction of the remaining intact cortex, medial collateral ligament, and patellar ligament [2]. The 200-N intervention-induced compressive load was uniformly exerted onto the distracted cortex of the tibial opening (Figure 3a). The physiological loads were estimated to be two times body weight (70 kgf) to simulate the compressive load on the knee during single limb stance [23]. In this study, the restoration of the physiological transfer of the knee load was assumed, thus leading to 40% and 60% of load repartition between the lateral and medial plateaus, respectively [24] (Figure 3a). As an illustration, the components of the physiological and surgical loads in terms of the four quadrants are given in Figure 3b. The degree-of-freedom of the nodes are totally fixed at the distal end of the tibia.

This study employed an automatic mesh generation algorithm with Simulation Version 2010 software (SolidWorks Corporation, Concord, MA, USA), which provides a special element density at plate-screw junctions three times that of the remainder of the model with the overall average element size of 2 mm. The meshing strategy was designed for curved element boundaries, thus there were no sharp discontinuities to induce an unrealistically high stress concentration. By using the aspect ratio and Jacobian checks, all elements were within acceptable distortion limits to maximize the result accuracy. The model was meshed by ten-node tetrahedral solid elements. On average, the final meshes consisted of 110,000 elements and 155,000 nodes for four finite-element models. The mesh refinement at the plate-screw interfaces was executed for modeling accuracy until excellent monotonic convergence behavior with < 1% difference in the total strain energy was achieved.

### Comparison indices

Four indices were chosen to compare the difference in stress and micromotion between the four plate variations. The first three were the indices of implant and bone failure that were calculated in terms of maximal von Mises stresses at screws, plates, and surrounding bone. The last was the index of construct stability for measuring the change in height at edges *aa*, *bb*, and *cc* of the opening (Figure 2b). The load ratio through the posterior to the anterior leg at the section plane *AA* was used to provide information of the load-transferring mechanism at the distal legs of the T+I and π plates (Figure 1c).

## Results

### Concentrated stresses of screw, plate, and bone

**Figure 4:**
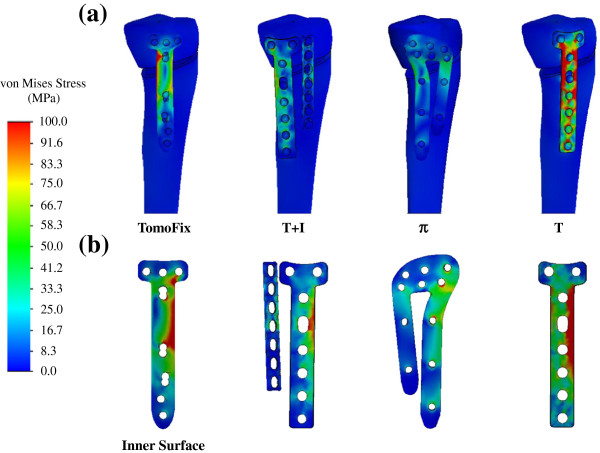
The stress distributions of the (a) four plate systems and (b) plate inner surfaces.

**Figure 5:**
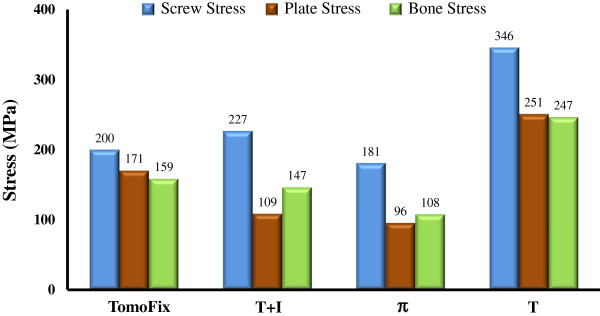
Maximal von Mises stresses of the screw, plate, and bone for the four plate systems.

The stress distributions of the four plate systems and the maximal stresses of the screw, plate, and bone are shown in Figures 4 and 5, respectively. In general, the medial opening highly stresses the implants, thus causing some screws and plate holes to become potential locations for failure by yielding and cracking. The screw stress of the T construct was the highest, followed by the T+I and TomoFix, and the π construct was the least stressed. Compared with the TomoFix construct, the screw stresses of the T+I and π constructs respectively increased 13.5% and decreased 9.5%. For the T+I construct, the addition of the I plate can significantly decrease screw stress by 34.4%.

For the plate stresses, the T construct was still the most stressed and was followed by the TomoFix. The plate stresses of the other constructs were comparable and 67.5% less than that of the TomoFix construct. Similar to the screw stress, the use of the I plate can decrease 56.6% of the plate stress. For the T-shaped design, the use of locking screws and titanium alloy made the plate stress of the TomoFix system 31.9% less than that of the T plate. The difference in bone stress between the four plate-bone constructs was similar to that of the differences between the stresses in the plates. The surrounding bone of the T plate was the highest stressed and the stress values were 55.3%, 68.0%, and 128.7% higher than the TomoFix, T+I, and π constructs, respectively. For the one-leg systems (*i.e.* TomoFix and T), the screw, plate, and bone stresses concentrated at the interfaces, and the most proximal screw below the opening, was the most stressed. The posterior leg of the two-leg system (*i.e.* T+I and π) was more loaded and also stressed highest at the same region as the one-leg design.

### Height change and load ratio

**Figure 6:**
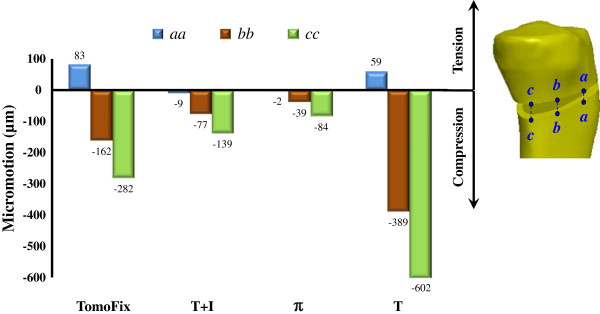
The wedge-edge micromotion of the four plate systems.

Along the three edges, the changes in the height of the medial opening are shown in Figure 6. For all plate systems, the opening wedge at the edge *cc* was compressed and the micromotion was the highest than the other edges. Nearest to the wedge tip, the micromotion at the edge *aa* was the least. At the edge *cc*, the micromotion of the T+I and π constructs were respectively reduced by 50.7% and 70.2% compared with the TomoFix. The hybrid use of the T and I plates can decrease micromotion by 76.9% compared to that of the T construct. Compared with the TomoFix and T plates, the two-leg design can stabilize the opening wedge at the edge *aa* and eliminate the undesired tension at the wedge tip. On average, this can reduce wedge-tip micromotion by 92.3% in the one-leg design. The load ratio was respectively 4.7 and 3.9 between the posterior and anterior legs of the T+I and π plates.

## Discussion

The knee is one of the most heavily loaded joints of the human body during daily activities. For the HTO surgery, the medial opening is an extremely unstable condition for the proximal tibia, and the fixation device is used to stabilize the opening and enhance bone union. This study used the one- and two-leg plates to evaluate the effects of locking screw and plate leg on the construct stress and wedge micromotion. For the one-leg design, the screw, plate, and bone stresses of the TomoFix construct were respectively 42.2%, 31.9%, and 35.6% less than those of the T construct (Figure 5). For the two-leg design, the aforementioned stresses of the π plate can be reduced by 20.3%, 11.9%, and 26.5% as compared with the T+I construct. This indicated that the use of locking screws can significantly reduce the mechanical demands of the implants and surrounding bone.

**Figure 7:**
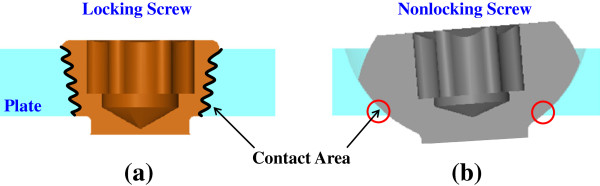
The schematic diagrams to show the difference in contact area between the locking and nonlocking screw-plate constructs: (a) Locking screw (b) Nonlocking screw.

The contact behavior at the plate-screw interfaces can be used to account for the effects of the locking screws (Figure 7). The head of the nonlocking screw can freely rotate within the plate hole but the threads of both the screw head and plate hole were tightly locked. Due to the intimate contact, the screw and plate stresses of the locking systems (*i.e.* TomoFix and π) could be more uniformly distributed and reduced (Figure 5) [25,26]. Upon loading, the nonlocking screw potentially rotates and makes line or even point contact with the plate hole (Figure 7b). This led to the highly concentrated stresses at the screw-plate interfaces of the nonlocking systems (*i.e.* T and T+I) [27]. The biomechanical study of Seide *et al.*[28] demonstrated that the stable fixation at the screw-plate interfaces can distribute the bony loads more uniformly and avert stress concentration at some local sites. This can be used to explain the reduction of bone stress of the TomoFix and π systems (Figure 5).

**Figure 8:**
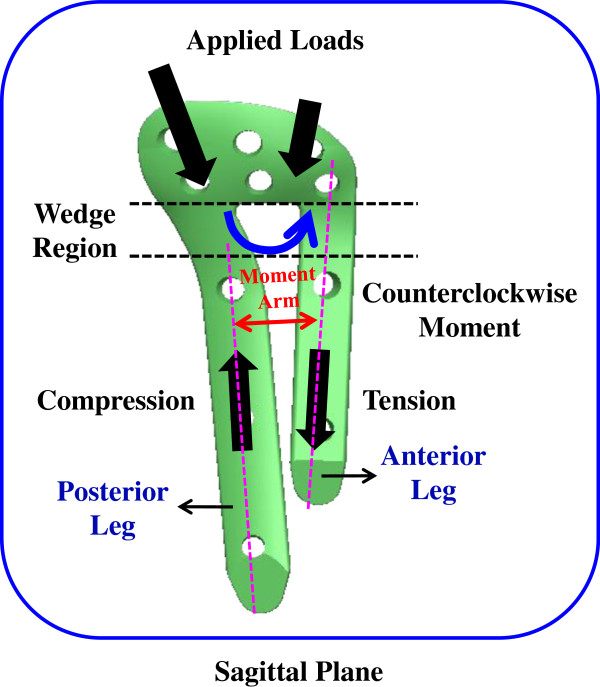
The legs of the π system develop an equivalent force-couple to resist the knee loads.

Two plate-leg factors enhanced the biomechanical performance of the HTO construct: more screws to support the knee loads and the wider supporting base below the opening. The two-leg system provides more screws to stabilize the medial opening, thus significantly decreasing the plate and bone stresses and suppressing the wedge micromotion (Figures 5 and 6). For the T+I and π systems, there are two nearly parallel legs to transmit the knee loads through the opening (Figure 1). The current authors hypothesized that the plate serves as a fulcrum to transmit the knee loads from the proximal to the distal bones. With respect to the sagittal plane, the nonuniform distribution of the knee loads potentially induced a counterclockwise moment to the tibial plateau (Figures 3b and 8). The original loads and induced moment were balanced by the plate and remaining cortex at the wedge tip (*i.e.* edge *cc*). The two plate legs can reconstruct a wider supporting base to behave as an effective force-couple mechanism. The anterior leg is mainly subject to the tension loads and the majority of the compression loads transmits through the posterior leg. The moment arm spanned by the legs can reduce the moment-induced stresses of bone and implants and wedge-tip micromotion (Figure 6).

For the two-leg systems, the load ratio of the posterior to anterior legs can provide biomechanical information of the load-transferring mechanism. If the tops of both T and I plates were linked, the value of the load ratio could be decreased from 4.7 to 3.9. This indicated that the anterior leg of the π plate could share the loads of the posterior leg to reduce the mechanical failure at that location. Meanwhile, the results of the load ratio demonstrated the effective distribution of the enormous loads from the knee joint to the distal tibia.

The height changes in the edges *aa*, *bb*, and *cc* were regarded as the indices of the construct stability. Except for the *π* plate, the micromotions of the other systems consistently exceeded the reported maximum value (> 100 μm) of the allowable movement for the bone union [29]. However, some studies have shown that the highly rigid fixation may cause osteoporosis due to the stress-shielding effect [30,31]. Moreover, adequate micromotion of fracture interfaces can enhance the callus formation [32,33]. Historically, the trade-off between rigid fixation and interfacial micromotion is still unknown [34].

For the one-leg systems, the wedge micromotions were significantly higher that the counterparts and were contributed to the bone-screw loosening and construct instability [2,35]. At the wedge tip (edge *aa*) of the one-leg systems, Figure 6 showed the occurrence of bone separation that results from the leverage effect and negatively affects the callus formation [36]. In the literature, some researchers proposed the anteromedial side as the optimal position of the one-leg plate to stabilize an osteotomized tibia [2,37]. On the other hand, follow-up and cadaveric studies of the opponents found that the anteromedial placement induces posterior tilt of the tibial plateau and construct instability [38,39]. Comparatively, the two-leg plate provides a greater area to cover the circumferential sides of the medial opening (Figure 1). This can deter the placement-induced effects of the one-leg plate on the construct responses and ensure more stable support to the opening.

There are some limitations inherent in this study. The use of both physiological and surgical loads may be overestimated for the early stage of the HTO healing. After surgery, however, the treated leg was often partially loaded by using a crutch or a walker [40]. If shorter bed-resting time was desired, this study can provide the worst-case comparison of the different HTO plate systems in the situation of the full body-weight [34]. From the biomechanical viewpoint, both bone variations and wedge sizes might alter the stabilizing effect of those HTO plates. However, these were not included in this study and the experimental validation should be performed.

## Conclusion

The optimal use of the HTO plates involves the trade-off between surgical convenience, construct stability, and the effects of stress-shielding. The incision size of a one-leg plate can be less than that of its counterpart. If the plate thickness was appropriately designed, the one-leg plate can shield the bony loads less than the two-leg system. However, surgical planning for plate placement is necessary due to the smaller size of the one-leg system. The two-leg plate can provide a stable plate-leg base and form a force-couple mechanism to effectively resist the applied loads. If the construct stability is the major concern, the two-leg system can be used for patients with a heavy load demand and greater proximal tibial size. Except for the larger incision size, however, the two-leg system may result in stress-shielding in the region around the wedge and smaller micromotion across the opening gap. It suggested the one-leg system with locking screws can be used for the majority of the patients without heavy bodyweight and poor bone quality. Alternatively, the two-leg plate is recommended to be used for the heavy (*i.e.* higher implant stress) and osteoporotic (*i.e.* weaker bone strength) patients.

## Competing interests

The authors declare that they have no competing interests.

## Authors’ contributions

CAL, SCL, and SYH conceived of the study, participated in the design of the study and performed the data analyses. CAL and SCL formulated the model and drafted the manuscript with the help of CMC and CST. All authors carried out the experiments, read, and approved the final manuscript.

## Pre-publication history

The pre-publication history for this paper can be accessed here:

http://www.biomedcentral.com/1471-2474/14/110/prepub

## References

[B1] Ryohei T Hiroyuki I Masato A Haruhiko B Izumi S Ken K Yasuhsi A Tomoyuki S Medial opening wedge high tibial osteotomy with early full weight bearing J Arth Rel Surg 2009 25 46 53 10.1016/j.arthro.2008.08.015 19111218

[B2] Blecha LD Zambelli PY Ramaniraka NA Bourban PE Manson JA Pioletti DP How plate positioning impacts the biomechanics of the open wedge tibial osteotomy: A finite element analysis Comp Meth Biomech Biomed Eng 2005 8 307 313 10.1080/10255840500322433 16298852

[B3] Coventry MB Osteotomy of the upper portion of the tibia for degenerative arthritis of the knee: a preliminary report J Bone Joint Surg Am 1965 47 984 990 14318636

[B4] Jackson JP Waugh W Green JP Wood H High tibial osteotomy for osteoarthritis of the knee J Bone Joint Surg Br 1969 51 88 94 5766363

[B5] Su CL Kwang AJ Chang HN Soong HJ Seung HH The short-term follow-up results of open wedge high tibial osteotomy with using an aescula open wedge plate and an allogenic bone graft: the minimum 1-year follow-up results Clin Orthop Surg 2010 2 47 54 10.4055/cios.2010.2.1.47 20191001 PMC2824095

[B6] Staubli AE Simoni CD Babst R Lobenhoffer P TomoFix: a new LCP-concept for open wedge osteotomy of the medial proximal tibia – early results in 92 cases Injury 2003 34 55 62 10.1016/j.injury.2003.09.025 14580986

[B7] Spahn G Wittig R Primary stability of various implants in tibial opening wedge osteotomy: a biomechanical study J Orthop Sci 2002 7 683 687 10.1007/s007760200121 12486473

[B8] Amendola A Bonasia DE Results of high tibial osteotomy: review of the literature Int Orthop 2010 34 115 160 10.1007/s00264-008-0574-3 19838706 PMC2899364

[B9] Agneskirchner JD Freiling D Hurschler C Lobenhoffer P Primary stability of four different implants for opening wedge high tibial osteotomy Knee Surg Sports Traumatol Arthrosc 2006 14 291 300 10.1007/s00167-005-0690-1 16284740

[B10] Stoffel K Stachowiak G Kuster M Open wedge high tibial osteotomy biomechanical investigation of the modified Arthrex Osteotomy Plate and the TomoFix Plate Clin Biomech 2004 19 944 950 10.1016/j.clinbiomech.2004.06.007 15475127

[B11] Nelissen EM Langelaan EJ Nelissen RGHH Stability of medial opening wedge high tibial osteotomy: a failure analysis Int Orthop 2010 34 217 223 10.1007/s00264-009-0723-3 19189104 PMC2899369

[B12] Spahn G Complications in high tibial (medial opening wedge) osteotomy Arch Orthop Trauma Surg 2004 124 649 653 10.1007/s00402-003-0588-7 14520581

[B13] Lobenhoffer P Agneskirchner JD Improvements in surgical technique of valgus high tibial osteotomy Knee Surg Sports Traumatol Arthrosc 2003 11 132 138 12774149 10.1007/s00167-002-0334-7

[B14] Kolb W Guhlmann H Windisch C Kolb K Koller H Grützner P Opening-wedge high tibial osteotomy with a locked low-profile plate J Bone Joint Surg Am 2009 91 2581 2588 10.2106/JBJS.H.01047 19884431

[B15] Zaki SH Rae PJ High tibial valgus osteotomy using the Tomofix plate-medium-term results in young patients Acta Orthop Belq 2009 75 360 367 19681323

[B16] Valkering KP van den Bekerom MP Kappelhoff FM Albers GH Complications after tomofix medial opening wedge high tibial osteotomy J Knee Surg 2009 22 218 225 10.1055/s-0030-1247752 19634725

[B17] Brosset T Pasquierb G Migaudb H Gougeon F Opening wedge high tibial osteotomy performed without filling the defect but with locking plate fixation (TomoFix™) and early weight-bearing: Prospective evaluation of bone union, precision and maintenance of correction in 51 cases Orthop Traumatol Surg Res 2011 97 705 711 10.1016/j.otsr.2011.06.011 22001198

[B18] Noyes FR Mayfield W Barber-Westin SD Albright JC Heckmann TP Opening wedge high tibial osteotomy: an operative technique and rehabilitation program to decrease complications and promote early union and function Am J Sports Med 2006 34 1262 1273 10.1177/0363546505286144 16493168

[B19] Noyes FR Barber-Westin SD Hewett TE High tibial osteotomy and ligament reconstruction for varus angulated anterior cruciate ligament-deficient knees Am J Sports Med 2000 28 282 296 10843117 10.1177/03635465000280030201

[B20] Hankemeier S Hufner T Wang G Kendoff D Zeichen J Zheng G Krettek C Navigated open-wedge high tibial osteotomy: advantages and disadvantages compared to the conventional technique in a cadaver study Knee Surg Sports Traumatol Arthrosc 2006 14 917 921 10.1007/s00167-006-0035-8 16501952

[B21] Hoffler CE Moore KE Kozloff K Zysset PK Goldstein SA Age, gender, and bone lamellae elastic moduli J Orthop Res 2000 18 432 437 10.1002/jor.1100180315 10937630

[B22] Reillya DT Burstein AH The elastic and ultimate properties of compact bone tissue J Biomech 1975 8 393 396 10.1016/0021-9290(75)90075-5 1206042

[B23] Taylor WR Heller MO Bergmann G Duda GN Tibio-femoral loading during human gait and stair climbing J Orthopaedic Res 2004 22 625 632 10.1016/j.orthres.2003.09.003 15099644

[B24] Johnson F Scarrow P Waugh W Assessments of loads in the knee joint Med Biol Eng Comp 1981 19 237 243 10.1007/BF02442721 7266106

[B25] Gueorguiev B Ockert B Schwieger K Wähnert D Lawson SM Windolf M Stoffel K Angular stability potentially permits fewer locking screws compared with conventional locking in intramedullary nailed distal tibia fractures: a biomechanical study J Orthop Trauma 2011 25 340 346 10.1097/BOT.0b013e3182163345 21577069

[B26] Eric JS Ran S Frederick K Kenneth AE The current status of locked plating: the good, the Bad, and the ugly J Orthop Trauma 2008 22 479 486 10.1097/BOT.0b013e31817996d6 18670289

[B27] Joseph BC Casey D David MW John MK Susan PJ Ross HP Comparison of the mechanical behaviors of locked and nonlocked plate/screw fixation applied to experimentally induced rotational osteotomies in canine ilia Vet Surg 2012 41 103 113 22092198 10.1111/j.1532-950X.2011.00913.x

[B28] Seide K Zierold W Wolter D Kortmann HR The effect of an angle-stable plate-screw connection and various screw diameters on the stability of plate osteosynthesis. An FE model study Unfallchirurg 1990 93 552 558 2281325

[B29] Pilliar RM Lee JM Maniatopoulos C Observations on the effect of movement on bone ingrowth into porous-surfaced implants Clin Orthop Relat Res 1986 208 108 113 3720113

[B30] Benli S Aksoy S HavItcIoglu H Kucuk M Evaluation of bone plate with low-stiffness material in terms of stress distribution J Biomech 2008 41 3229 3235 10.1016/j.jbiomech.2008.08.003 18805533

[B31] Fan Y Xiu K Duan H Zhang M Biomechanical and histological evaluation of the application of biodegradable poly-L-lactic cushion to the plate internal fixation for bone fracture healing Clin Biochem 2008 23 S7 16 10.1016/j.clinbiomech.2008.01.005 18291564

[B32] Augat P Burger J Schorlemmer S Henke T Peraus M Claes L Shear movement at the fracture site delays healing in a diaphyseal fracture model J Orthop Res 2003 21 1011 1017 10.1016/S0736-0266(03)00098-6 14554213

[B33] Noordeen MH Lavy CB Shergill NS Tuite JD Jackson AM Cyclical micromovement and fracture healing J Bone Joint Surg Br 1995 77 645 648 7615614

[B34] Raja Izaham RM Abdul Kadir MR Abdul Rashid AH Hossain MG Kamarul T Finite element analysis of Puddu and Tomofix plate fixation for open wedge high tibial osteotomy Injury 2012 43 898 902 10.1016/j.injury.2011.12.006 22204773

[B35] KazImoglu C Akdogan Y Sener M Kurtulmus A KarapInar H Uzun B Which is the best fixation method for lateral cortex disruption in the medial open wedge high tibial osteotomy? A biomechanical study Knee 2008 15 305 308 10.1016/j.knee.2008.04.004 18539033

[B36] Sato H Morishita S Effect of quadriceps exercise on synostosis following tibial osteotomy with internal fixation: a finite element simulation Clin Biomech 1999 14 1 6 10.1016/S0268-0033(98)00028-X 10619084

[B37] Koshino T Murase T Saito T Medial opening-wedge high tibial osteotomy with use of porous hydroxyapatite to treat medial compartment osteoarthritis of the knee J Bone Joint Surg Am 2003 85A 78 85 12533576 10.2106/00004623-200301000-00013

[B38] Rodner CM Adams DJ Diaz-Doran V Tate JP Santangelo SA Mazzocca AD Arciero RA Medial opening wedge tibial osteotomy and the sagittal plane: the effect of increasing tibial slope on tibiofemoral contact pressure Am J Sports Med 2006 34 1431 1441 10.1177/0363546506287297 16636350

[B39] Christoph BM Emanuel G Stefan WW Roland PJ Accuracy of frontal and sagittal plane correction in open-wedge high tibial osteotomy J Arthros Rel Surg 2004 20 366 372 10.1016/j.arthro.2004.01.024 15067275

[B40] Kim SH Chang SH Jung HJ The finite element analysis of a fractured tibia applied by composite bone plates considering contact conditions and time-varying properties of curing tissues Compos Struct 2010 92 2109 2118 10.1016/j.compstruct.2009.09.051

